# *Lacticaseibacillus paracasei* INIA P708: a safe human isolate with probiotic and technological potential

**DOI:** 10.1007/s00253-026-13984-x

**Published:** 2026-08-04

**Authors:** Eduardo D. Lozano-Escobar, Eva Rodríguez-Mínguez, Karel H. M. van Wely, Ángela Peirotén, Modesto Redrejo-Rodríguez, Antonia Picon

**Affiliations:** 1 Departamento de Tecnología de Alimentos, Instituto Nacional de Investigación y Tecnología Agraria y Alimentaria (INIA), CSIC, Carretera de La Coruña Km 7, Madrid, 28040 Spain; 2 Instituto de Investigaciones Biomédicas Sols-Morreale (UAM-CSIC) and Departamento de Bioquimica, Universidad Autónoma de Madrid (UAM), Arturo Duperier 4, Madrid, 28029 Spain; 3 Department of Immunology and Oncology, Centro Nacional de Biotecnología (CNB), CSIC, Darwin 3, Campus UAM Cantoblanco, Madrid, 28049 Spain

**Keywords:** *Lacticaseibacillus paracasei*, Probiotic potential, Ropy, Safety evaluation, Genome analysis, Thiamine

## Abstract

**Abstract:**

*Lacticaseibacillus paracasei* INIA P708 was isolated from breast-fed infant feces. It stood out by its stable ropy phenotype, which could indicate exopolysaccharide (EPS) production and antimicrobial properties. Bearing in mind its potential use in the food chain, the objective of this work was to study *L. paracasei* INIA P708 potential probiotic traits, and technological and safety characteristics, in comparison to the commercial probiotic *L. rhamnosus* GG strain. A full genomic analysis was also performed to comply with EFSA requirement for microorganisms intentionally added to the food chain. EPS production was tested by confocal scanning laser microscopy (CSLM), labelling bacterial cultures with fluorescent lectin conjugates. As probiotic traits, *in vitro* survival under major gastrointestinal conditions, biofilm formation, and antimicrobial activity was tested. As technological properties, survival as lyophilized cultures, growth in milk, proteolytic and acidifying properties, salt tolerance, and growth with prebiotic compounds were studied. As safety traits, antibiotic resistance, biogenic amine production, and mucine degradation were also tested. The complete, closed, INIA P708 genome sequence was determined by combining long and short reads. CSLM confirmed EPS production by *L. paracasei* INIA P708, and their attachment to the cell surface. INIA P708 showed good survival rates after lysozyme exposure (98%) and after simulated major GIT conditions (97%). However, under the tested conditions, and opposite to what we observed for GG, INIA P708 was not able to form biofilm. Both strains showed a clear inhibition halo against *Listeria monocytogenes* and a diffuse halo against *Klebsiella oxytoca*. The lyophilization process lowered levels by 0.2–0.3 log units for both strains, without changes after 21 days of refrigerated storage. Salt had a significant effect on the growth of both strains, with INIA P708 being more sensitive to salt than GG. Regarding safety traits, both strains were susceptible to chloramphenicol, erythromycin, rifampicin, and tetracycline and resistant to kanamycin and vancomycin. They were neither able to produce biogenic amines from histidine or tyrosine nor to degrade mucine. The closed INIA P708 genome is composed by a chromosome (3.08 Mpb) and a plasmid (66.8 Kpb), with a GC content of 46.3%. Detailed genome analysis confirmed *L. paracasei* INIA P708 identification and safety, supported its use as an industrial strain, and revealed new potential probiotic traits in this strain.

**Key points:**

• *Lacticaseibacillus paracasei INIA P708 is a human isolate with high potential.*

• *It might be used in health-promoting and/or in technological applications.*

• *Full genome resolution confirms it could be safely used as an industrial strain.*

**Supplementary information:**

The online version contains supplementary material available at https://doi.org/10.1007/s00253-026-13984-x.

## Introduction

The *Lacticaseibacillus* genus is one of the 23 novel genera created after reclassification of the old *Lactobacillus* genus (Zheng et al. 2020). It includes all strains of the previous *Lactobacillus casei* group. Strains of the *Lacticaseibacillus* genus are non-motile rods, oxidase negative, and homofermentative (often producing D(−)- and L(+)-lactic acid from glucose). Some species are able to metabolize pentoses via the phosphoketolase pathway. Temperature range is never below 10 °C or above 45 °C (Zheng et al. 2020). They are members of the normal human and animal gut microbiota and are extensively used in the food industry as dairy starters and also as bacteria with probiotic features (Smokvina et al. 2013). They are among the first bacteria to colonize the infant gut and are considered health-promoting agents present in human breast milk (Ruiz, et al. 2019).

*Lacticaseibacillus paracasei* INIA P708 belongs to the bacterial culture collection of the Department of Food Technology at the National Institute for Agricultural and Food Research and Technology (INIA-CSIC) and was isolated from breast-fed infant feces (Rodríguez et al. 2012). As it possesses a stable ropy phenotype, it may produce exopolysaccharides (EPS). EPS-producing strains are in high demand. In probiotic use, EPS confer health-promoting properties as intestinal microbiota modulation, cholesterol level reduction, or immunomodulatory and/or antitumoral effects, among others (Ale et al. 2020; Oerlemans et al. 2021; Oleksy and Klewicka 2018). In dairy products, *in situ* EPS production improves rheological properties, prevents syneresis, and acts as an emulsion stabilizing agent, avoiding the use of additives (Bengoa et al. 2023). However, strains with health-promoting properties are frequently unable to withstand the conditions used in food production, not reaching sufficient viable numbers (6–7 log CFU/mL) for sustaining their therapeutic effects (Ranadheera et al. 2010). Therefore, to achieve application in the food industry, it is important to test not only the probiotic characteristics (e.g., survival under gastrointestinal conditions, biofilm formation, antimicrobial activity) of a certain strain, but also its technological abilities (e.g., survival as lyophilizate, growth in milk, proteolytic and acidifying activities, salt tolerance).

According to the European Food Safety Authority (EFSA), *L. paracasei* strains are included in the list of microorganisms with “Qualified presumption of safety” (QPS) status (Hazards et al. 2025). However, since March 2021, EFSA requires a genomic analysis for microorganisms intentionally added to the food chain (European Food Safety Authority 2024).

The objective of this work was to study potential probiotic traits and the technological characteristics of the *L. paracasei* INIA P708 strain, and ensure its safety. To achieve the full resolution of bacterial genome and delineation of plasmids, a combination of short- and long-read sequencing methods was applied. Thus, the *in vitro* experiments, together with the whole genome sequence and *in silico* analysis, allowed us to delve into the probiotic and technological characteristics of this strain.

## Materials and methods

### Bacterial strains

*L. paracasei* INIA P708 was isolated from healthy breast-fed infant feces at the INIA lab facilities (Madrid), using Rogosa (Difco, Beckton, Dickinson and Co, Le Pont de Claix, France) agar plates supplemented with 10 µg/mL vancomycin (Merck, St. Louis, MO, USA). Plates were incubated at 37 °C for 48 h under anaerobic conditions (AnaeroGen, Oxoid, Basingstoke, UK). It was routinely cultured in de Man, Rogosa, and Sharpe (MRS, Oxoid) broth, at 37 °C for 48 h in an anaerobic atmosphere, and stored at −80 °C in MRS with 5% (v/v) glycerol. Isolates were subcultured twice on MRS before their use in subsequent experiments. INIA P708 belongs to the INIA beneficial culture colection of the INIA (CSIC) Department of Food Technology.

The commercial probiotic *Lacticaseibacillus rhamnosus* GG strain (Doron et al. 2005), included for comparative purposes, was conserved and grown under the same conditions.

### Ropy phenotype and EPS visualization

The characteristic “ropy” phenotype was checked by the formation of a long filament when a loop was introduced into the colony (Hidalgo-Cantabrana et al. 2013).

To visualize EPS production, bacterial cultures were labelled with lectins that recognize different sugar moieties. Briefly, INIA P708 and GG were grown overnight in MRS, and 100 µl samples were spotted on coverslips previously coated with poly-L-lysine (Merck). Cultures were fixed by the addition of an equal volume of 7.5% formaldehyde (Merck) in PBS and incubation at room temperature for 15 min. The coverslips were then washed twice with PBS for 5 min, incubated for 15 min with a PBS solution containing 50 µg/ml concanavalin A (ConA) - Alexa Fluor™ 488 conjugate (Life Technology Corporation, Eugene, OR, USA), and 50 µg/ml wheat germ agglutinin (WGA) - Alexa Fluor™ 555 conjugate (Life Technology Corporation), and washed briefly three times with PBS. Coverslips were washed twice with PBS for 5 min and incubated with a 5 µg/mL Hoechst 33342 solution (Merck) for 5 min, protected from light. Finally, coverslips were drained and mounted on ProLong™ Gold antifade mountant (Life Technology Corporation).

Images were captured on an IX81 laser scanning confocal microscope (Olympus, Tokyo, Japan), using serial illumination with 405, 488, and 543 nm excitation wavelengths. RGB color images were taken using a 63x objective with a numerical aperture of 1.40 and 4.0 (wide) and 12.0 (close-up) zoom factors. Images were processed in ImageJ (Collins 2007). For deconvolution of close-up images, the iterative deconvolve 3D plugin was used (Dougherty 2005).

### Probiotic traits

#### Survival under major gastrointestinal conditions

Lysozyme sensitivity and survival to simulated major gastrointestinal conditions were tested as previously described (Rodríguez et al. 2012). Briefly, after inoculation in fresh broth with or without 25 µL/mL lysozyme, cultures were incubated at 37 °C for 5 min and plated on MRS agar. Viable counts were determined after 48 h incubation under anaerobic conditions in control and treated cultures.

For simulated major gastrointestinal stresses, 1 mL of cell suspensions in skim milk was mixed with 9 mL of artificial gastric juice (prepared as described (Zarate et al. 2000) and incubated at 37 °C for 1 h under anaerobic conditions. Then, a 1 mL-sample of the mixture was added to 9 mL of bile solution (0.15% ox-bile desiccated, Oxoid) and kept at the same conditions for 1 h, before enumeration.

Both tests were carried out in duplicate.

#### Biofilm formation

Experiments were performed in 96-multiwell plates (Thermo Fisher Scientific, Roskilde, Denmark), as previously described (Lee et al. 2019; Rodríguez-Mínguez et al. 2024). Individual wells were filled with 200 µL of Brain Heart Infusion broth (BHI, Oxoid) containing 0.05% cysteine-HCl and 0.02% oxgall (Merck) and inoculated with 2 µL of bacterial suspensions (cells grown on plates were suspended in sterile 0.2% peptone water with 0.05% cysteine-HCl, approx. 8.5 log CFU/mL). Six replicates per strain were prepared in two independent experiments. Non-inoculated wells were included as negative controls. After 24 h of anaerobic incubation at 37 °C, wells were emptied, filled with a 0.4% crystal violet solution, incubated for 30 min, and washed three times with distilled water, and 30% glacial acetic acid was applied to dissolve the formed biofilms. Plates were introduced in a microplate reader (Multiskan Spectrum, Thermo Fisher Scientific Oy, Vantaa, Finland), and absorbance at 600 nm (A_600_) was measured.

#### Antimicrobial activity

The antimicrobial activity of strains INIA P708 and GG was studied on four pathogenic strains of the INIA collection in a similar way as previously described (Rodríguez-Mínguez et al. 2024). Gram-positive strains *Clostridium perfringens* CECT 486 and *Listeria monocytogenes* Scott A CECT 5672 and Gram-negative strains *Klebsiella oxytoca* INIA Col 108 and *Salmonella enterica* serovar Enteritidis CECT 4155 were grown in BHI, at 37 °C for 24 h, under anaerobic conditions (*Clostridium perfringens* CECT 486), or as stand cultures. INIA P708 or GG cells grown on BHI plates were suspended in sterile 0.2% peptone water with 0.05% cysteine-HCl (approx. 8.5 log CFU/mL), and drops (5 µL) were deposited, in duplicate, on BHI agar plates. After anaerobic incubation at 37 °C for 24 h, a second layer of a 50 °C-tempered BHI soft agar, previously inoculated with fresh cultures of each pathogenic strain (at a 0.1%, final concentration of approx. 5.5–6 log CFU/mL), was poured. After incubation (24 h at 37 °C), plates were examined for the presence of inhibition halos.

The antimicrobial activity of strains INIA P708 and GG was also studied after growth in milk against *Salmonella enterica* serovar Enteritidis CECT 4155. Cell suspensions from freshly grown cultures of strains INIA P708 and GG were prepared in milk (approx. 9.0 log CFU/mL) and inoculated at 1% in milk, and cultures were growth at 37 °C for 24 h under anaerobic conditions. Non-inoculated milk served as control. Thereafter, cultures were inoculated with a *S. enterica* cell suspension (final concentration, approx. 4.0 log CFU/mL) and incubated at 37 °C for 24 h. Samples for microbial enumeration and pH determination were taken. All experiments in this section were performed in duplicate.

### Technological properties

#### Survival as lyophilized cultures

After collecting cells grown in MRS by centrifugation (6000 g for 5 min), they were resuspended in 10% reconstituted skim milk and frozen at −80 °C for 24 h. Subsequently, cultures were lyophilized in a Cryodos lyophilizer (Telstar S.A, Terrasa, Spain) operating at 1 Pa pressure and −45 °C for 24 h. Lyophilized cultures were then stored at 5 °C for 21 days. Freeze-dried cells were reconstituted using sterile water, and viability was determined by plate counting on MRS agar.

#### Growth in milk and refrigerated storage

The ability to grow in milk was tested in reconstituted skim milk (10% w/v, Oxoid) in two independent trials. After growth in MRS, cells were collected by centrifugation (6000 g for 5 min), resuspended in a sterile 0.1% peptone solution, and inoculated at 1% in reconstituted skim milk tubes. Cultures were incubated at 37 °C for 24 h under anaerobic conditions. Counts were assessed in duplicate on MRS agar at 0 and 24 h.

After incubation, milk cultures were stored at 5 °C. Viable cell population was determined after 14, 21, and 28 days, by plate counting on MRS agar, as previously described.

#### Proteolytic and acidifying activities

Proteolytic and acidifying activities were tested by growing in 10% reconstituted skim milk prior to inoculation at 2% (v/v) in 10 mL of 10% reconstituted milk and incubated at 37 °C for 24 h in anaerobic conditions. Proteolysis was determined on duplicate samples by the *o*-phthaldialdehyde (OPA) test (Church et al. 1983). The pH values were measured in duplicate, at 0 and 24 h, with a Crison pH-meter (model Basic 20; Crison Instruments, Barcelona, Spain).

#### Salt tolerance

Salt tolerance was tested by inoculating, at 1% and two different inocula (7 and 5 log CFU/mL), in MRS broth with 0, 1, 2, 3, 4, and 5% NaCl (Merck, St. Louis, MI, USA) and incubating at 37 °C for 24 h under anaerobic conditions. A_600_ was determined using a Beckman DU 650 spectrophotometer (Beckman Instruments SA, Madrid, Spain).

#### Growth in milk supplemented with prebiotic compounds

Growth in UHT skim milk supplemented with several prebiotic compounds, at 1%, was tested in triplicate. Reconstituted milk without prebiotic supplementation served as a control. Lactulose (Merck), fructooligosaccharides (FOS, BENEO P95, Beneo, Mannheim, Germany), and galactooligosaccharides (GOS, Clasado Bioscience, Reading, UK) were tested. Stock solutions were prepared at 30% (w/v) in demineralized water, filter sterilized, and stored at room temperature. Prior to inoculation, strains were cultured twice in MRS broth and once in UHT skim milk. The different milk preparations were inoculated at 1% (v/v) at a final concentration of approx. 5.5 log CFU/mL. Incubation was performed under anaerobic conditions at 37 °C for 24 h. Bacterial counts were determined on MRS agar at 0 h and 24 h.

### Safety traits

#### Antibiotic resistance

The susceptibility to clinically relevant antibiotics was semi-quantitatively screened by the disk diffusion method. Antibiotics tested included chloramphenicol (30 µg), erythromycin (15 µg), kanamycin (30 µg), rifampicin (5 µg), tetracycline (30 µg), vancomycin (30 µg), and sulphamethoxazole (50 µg). Commercial antibiotic disks (Liofilchem s.r.l, Teramo, Italy) were placed on BHI agar plates inoculated with a McFarland 0.5 standard of each strain. Plates were incubated at 37 °C in anaerobic conditions. After 48 h, inhibition halo diameters were measured (in mm, means ± SD of 3 trials) and interpreted as susceptible (S) or resistant (R).

#### Biogenic amine production

Decarboxylase activity was tested as previously described (Bover-Cid and Holzapfel 1999). The decarboxylase agar medium contained histidine or tyrosine (10 g/L each) as precursors and bromocresol purple as indicator. The histamine-producer *L. buchneri* St2A and the tyramine-producer *E. faecalis* INIA 4 were included in the assays as positive controls (Joosten et al. 1995).

#### Mucin degradation

Following the procedure described by Zhou et al. (2001), partially purified hog gastric mucin type III (HGM) (Sigma-Aldrich) was used to determine mucin degradation.

### Statistical analyses

Growth data were subjected to one-way analysis of variance (ANOVA) using the SPSS 29.0 statistical package (IBM Corporation, Armonk, NY, USA). Means were compared by Dunnett or Tukey tests.

### Whole genome sequencing and assembly

The whole-genome sequencing of *Lacticaseibacillus paracasei* INIA P708 was achieved by using both short- and long-read technologies. Short reads were obtained using the Illumina MiSeq platform (Illumina, California, USA) at the UCM Genomics Unit (Madrid, Spain), generating 150 bp paired-end libraries. Long reads were obtained from a Sequel-II PacBio system (Pacific Biosciences, California, USA) at the FISABIO sequencing facility (Valencia, Spain).

Illumina reads were analyzed using FastQC v0.12.1 (Andrews 2010) and processed with Trimmomatic v0.39 (Bolger et al. 2014). Adapters and low-quality reads were trimmed using the parameters ILLUMINACLIP:TruSeq3-PE.fa:2:30:10 SLIDINGWINDOW:4:20 LEADING:20 TRAILING:20 AVGQUAL:20 MINLEN:50. PacBio reads were analyzed with NanoPlot v1.42.0 (De Coster and Rademakers 2023). Finally, reads were decontaminated to remove sequences from human (GCF_000001405.40) and ΦX174 bacteriophage (GCF_000819615.1) sources, using Bowtie2 v2.5.3 (Langmead and Salzberg 2012). The *de novo* hybrid genome assembly was performed using Unicycler v0.5.1 (Wick et al. 2017) with default options. Assembly quality was evaluated using QUAST v5.2.0 (Gurevich et al. 2013) and CheckM2 v1.0.1 (Chklovski et al. 2023).

### Strain identification

The species was determined by calculating the Average Nucleotide Identity (ANI) using FastANI v1.33 (Jain et al. 2018) against publicly available *Lacticaseibacillus* genomes deposited in GenBank. In addition, the Type Strain Genome Server (TYGS) web server (Meier-Kolthoff and Göker 2019) was used to estimate digital DNA-DNA hybridization (dDDH). To confirm bacterial species, thresholds of 95% for ANI and 70% for dDDH were applied, as previously established by other authors (Chun et al. 2018; Richter and Rosselló-Móra 2009) and recommended by EFSA (2024).

### Genomic annotation

Structural genome annotation was performed using Bakta v1.9.3 (Schwengers et al. 2021) and version 5.1 of its database with default settings. Functional annotation was conducted based on protein sequences obtained from Bakta using the eggNOG-mapper v2.1.12 web server (Cantalapiedra et al. 2021) and the eggNOG5 database, with default settings. Terms related to COG (Clusters of Orthologous Groups), KEGG (Kyoto Encyclopedia of Genes and Genomes), and CAZy (Carbohydrate-Active Enzymes) were retrieved during this process.

### Mobile genetic elements and defense systems

Mobile genetic elements (MGEs), such as plasmids or bacteriophages, are DNA segments that encode enzymes and other proteins facilitating the transfer of genetic material between bacteria. The identification of these elements was performed using the end-to-end pipeline of geNomad v1.8.0 (Camargo et al. 2024), which runs a series of sequential modules enabling the detection of plasmids and viruses by combining traditional marker-based classification with a more advanced neural network-based approach. Additionally, the enable-score-calibration option was selected to estimate the probabilities of the recorded sequences being mobile genetic elements (MGEs). This methodology was complemented with widely used tools for plasmid and bacteriophage detection. Plasmid replicons were typed using PlasmidFinder v2.1.6 (Carattoli et al. 2014) with coverage and identity thresholds of 40% and 70%, respectively, while bacteriophages were detected through the PHASTEST (PHAge Search Tool with Enhanced Sequence Translation) web server (Wishart et al. 2023). To harmonize the results of prophage prediction from geNomad and PHASTEST, CheckV v1.0.3 (Nayfach et al. 2021) was used to filter out low-confidence regions, retaining only medium- and high-quality regions. Subsequently, the most similar viral sequences were searched using BLAST in the nt database.

Bacteria possess various defense systems to protect themselves against different types of MGEs. One of the most important, due to its applications in genome editing, is the CRISPR/Cas system (Jinek et al. 2012). Defense system identification was performed using the default settings of PADLOC v2.0.0 (Payne et al. 2021) and CRISPRCasTyper v1.8.0 (Russel et al. 2020).

### Detection of potentially adverse genes

Genes associated with antimicrobial resistance (AMR) were identified using the default settings of ResFinder v4.4.2 (Bortolaia et al. 2020), AMRFinderPlus v3.12.8 (Feldgarden et al. 2021), and RGI-CARD v6.0.3 (Alcock et al. 2023). The results from AMRFinderPlus were obtained through Bakta, as it runs this tool in the background for genomic annotation.

Virulence factors were identified using VirulenceFinder v2.0.4 (Kleinheinz et al. 2014) and BLAST alignments of gene and protein sequences against the VFDB database (Liu et al. 2022). In both cases, identity and coverage thresholds of 80% and 70%, respectively, were applied relative to the references.

The pathogenicity of the strain was evaluated using the machine learning model WSPC v0.0.6 (Naor-Hoffmann et al. 2022) and the PathogenFinder v1.1 web server (Cosentino et al. 2013). For the former, PGFam annotations were retrieved following the authors’ instructions and analyzed in prediction mode. For the latter, the All model was employed, as it is recommended for assessing the presence of specific pathogenic traits across most bacterial taxa.

### *In silico* analysis of antimicrobial potential

Genes associated with antimicrobial activities were analyzed using antiSMASH v7.1.0 (Blin et al. 2023), with detection strictness loose, which identifies biosynthetic gene clusters (BGCs) involved in the production of secondary metabolites, and the BAGEL4 web server (van Heel et al. 2018), which detects gene clusters related to the biosynthesis of ribosomally synthesized and post-translationally modified peptides (RiPPs) and bacteriocins.

### Genetic data availability

Sequence data were deposited at the NCBI under BioProject number PRJNA1401546. This Whole Genome Shotgun project has been deposited at DDBJ/ENA/GenBank under the accession JBZFHG000000000. The version described in this paper is version JBZFHG010000000.

## Results

### Ropy phenotype and EPS visualization

**Fig. 1:**
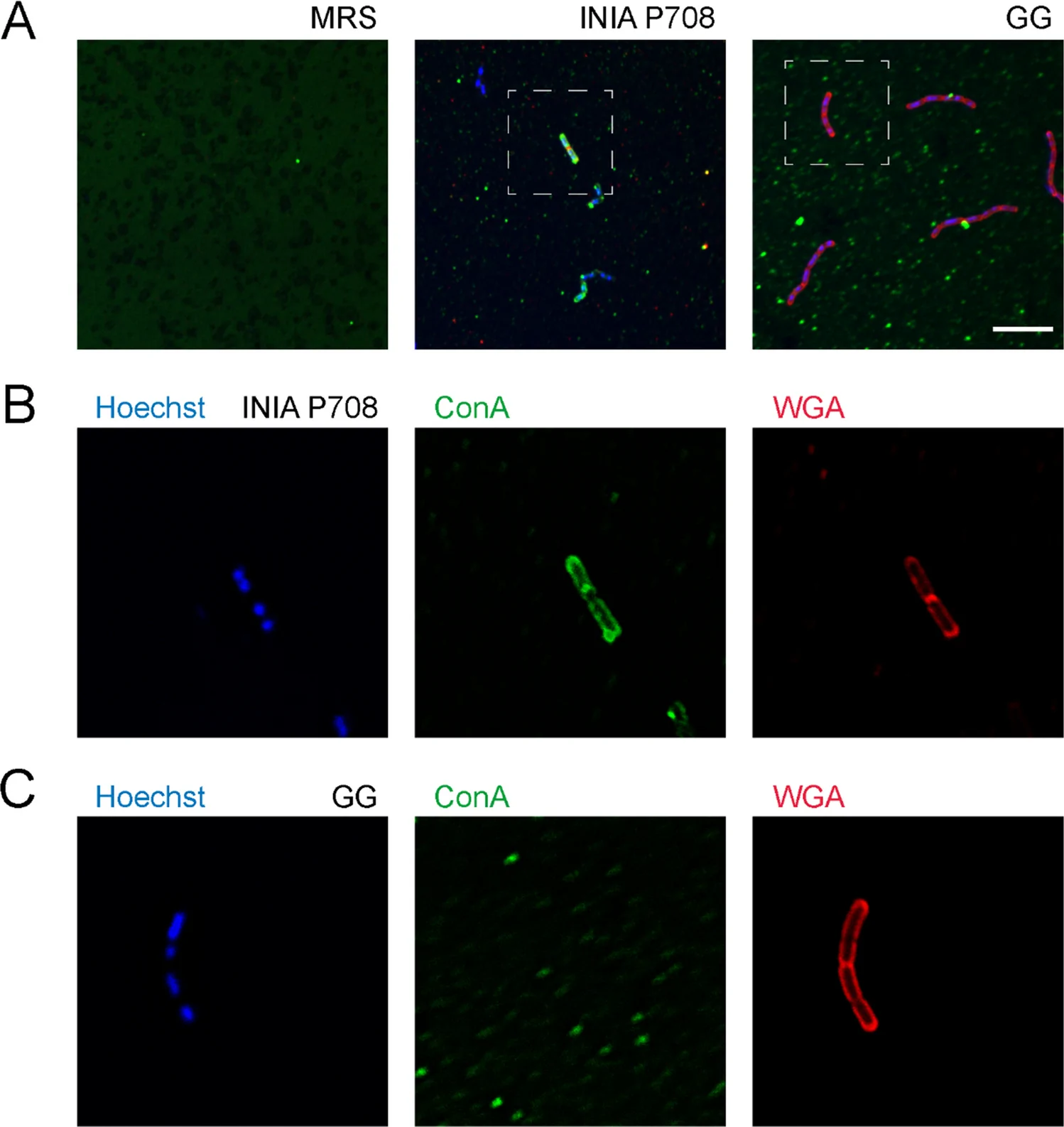
Exopolysaccharide visualization. Confocal scanning laser micrographs showing (from left to right): **A** the non-inoculated MRS medium, the *L. paracasei* INIA P708 cells, and the *L. rhamnosus* GG cells labelled with Hoechst 33,342 (blue), concanavalin A conjugate (ConA, green), and wheat germ agglutinin conjugate (WGA, red). The scale bar is 10 µm in length. **B** Close-up (threefold zoom) of the square depicted in the *L. paracasei* INIA P708 A panel, showing the images obtained for each labelling compound. **C** Close-up (threefold zoom) of the square depicted in the *L. rhamnosus* GG A panel, showing the images obtained for each labelling compound. Note the association of the ConA label with the cell surface of *L. paracasei* INIA P708, but its release into the medium in the *L. rhamnosus* GG culture.

EPS-producing strains are of interest due to the EPS dual function, as health-promoting substances and food additives (Llamas-Arriba et al. 2019). Because one of the indications of EPS production is a ropy phenotype, we tested whether INIA P708 colonies formed long strands when extended with an inoculation loop. While this characteristic was observed in INIA P708, it was not found for strain GG (Figure S1). To study the nature of EPS production in more detail, we subjected both strains to staining with ConA and WGA lectins. While ConA recognizes saccharide chains containing mannose and glucose, WGA is specific for N-acetyl-D-glucosamine and its derivatives (Strathmann et al. 2002). Subsequent evaluation of INIA P708 by confocal microscopy showed signals for both ConA and WGA (Fig. 1A). In contrast, GG controls (Francius et al. 2009) were labelled intensely by WGA but showed no cell-associated ConA signal. In addition to the cell-associated label, both strains showed a dotted pattern in the surrounding medium, whereas only trace amounts of staining were observed in uninoculated MRS controls. At higher magnification, the cell-associated lectins showed a distribution characteristic for the cell wall (Fig. 1B, C). Taken together, these results indicate robust EPS production by INIA P708.

### Probiotic traits

Bacteria colonizing the gut require molecular mechanisms conferring protection against the encountered deleterious conditions. Therefore, probiotic candidate strains need to prove resistance to the gastrointestinal tract (GIT) extreme conditions (Rodrigo-Torres et al. 2022). *L. paracasei* INIA P708 showed good survival rates after lysozyme exposure (98%) and after simulated major GIT conditions (97%) (Table 1).

**Table 1:** Probiotic traits of the studied strains: (1) microbial levels before and after exposure to lysozyme (L) and major gastrointestinal tract conditions (MGIC), (2) biofilm formation in medium BHIC with 0.02% oxgall (BHICO), and (3) antimicrobial activity against pathogenic strains in BHI agar plates.

Probiotic traits	Strains	
	INIA P708	GG
Microbial levels^1^		
Before L	9.40 ± 0.04 ^a^	8.35 ± 0.09 ^a^
After L	9.23 ± 0.05 ^b^	8.07 ± 0.10 ^b^
Before MGIC	9.45 ± 0.05 ^a^	8.31 ± 0.14 ^a^
After MGIC	9.19 ± 0.06 ^b^	8.11 ± 0.13 ^a^
Biofilm formation^2^	0.01 ± 0.01 ^b^	1.40 ± 0.09 ^a^
Antimicrobial activity^3,4^		
C	−	−
L	+	+
K	D	D
S	−	−

^1^Microbial levels were expressed as log CFU/mL. Means for the same strain with different superscripts differ significantly at *P* < 0.01

^2^Values were expressed as mean ± standard deviation of sextuplicate determinations in two experiments (*n* = 12). Means with different superscripts differ significantly at *P* < 0.01

^3^C = *C. perfringens* CECT 486; L = *L. monocytogenes* Scott A CECT 5672; K = *K. oxytoca* INIA Col 108; S = *S. enterica* serovar Enteritidis CECT 4155

^4^Codes: −  = no halo, D = diffuse halo; +  = halo < 7 mm; +  +  = halo 7–10 mm; +  +  +  = halo > 10 mm

Biofilm formation also could be an interesting trend for probiotic strains (Terraf et al. 2012). However, under the tested conditions, and opposite to what we observed for GG, INIA P708 was not able to form biofilm (Table 1).

The synthesis of antimicrobial substances is an important probiotic trait to inhibit pathogenic bacteria (Rodríguez et al. 2012). When antimicrobial activity was tested, both strains showed similar characteristics in plate cultures (Table 1): a clear inhibition halo against *L. monocytogenes* and a diffuse halo against *K. oxytoca* (Figure S2). However, in milk cultures against *S. enterica*, strain INIA P708 showed a stronger antimicrobial activity, as it completely inhibited *S. enterica* growth, whereas only a 2 log reduction was recorded for strain GG (Table 2).

**Table 2:** Microbial levels of *S. enterica* serovar Enteritidis CECT 4155 (S) grown in milk (at 37 °C for 24 h) as individual culture, or in INIA P708 or GG milk cultures (previously incubated at 37 °C for 24 h under anaerobic conditions).

Culture	Growth in milk	Growth in milk	S growth in milk/culture	
	0 h	24 h	0 h	24 h
Non-inoculated			3.99 ± 0.00	9.42 ± 0.01
INIA P708	7.28 ± 0.09	9.53 ± 0.02	2.83 ± 0.04	2.33 ± 0.17
GG	7.79 ± 0.01	7.74 ± 0.09	2.96 ± 0.04	7.83 ± 0.03

Values were expressed as mean ± standard deviation of duplicate determinations in two experiments (*n* = 4)

### Technological properties

Strains intended to be used in the food industry must be able to withstand the conditions used in food production (Ranadheera et al. 2010). Therefore, it is very important to test their technological properties. The lyophilization process lowered levels by 0.2–0.3 log units for both strains (Table 3), but no change was recorded after 21 days of refrigerated storage.

**Table 3:** Technological traits of the studied strains: (1) lyophilization survival, after the process and after storage at 5 °C, (2) microbial levels after incubation (at 37 °C for 24 h under anaerobic conditions) in milk, and after refrigerated storage at 5 °C, and (3) overall proteolysis and acidifying activity in milk cultures, incubated at 37 °C for 24 h under anaerobic conditions.

Technological traits	Non-inoculated	Strains	
		INIA P708	GG
Lyophilization survival^1^			
Stock		9.93 ± 0.02 ^a^	9.91 ± 0.08 ^a^
Lyophilizate 0 d		9.59 ± 0.09 ^b^	9.79 ± 0.07 ^b^
Lyophilizate 21 d		9.67 ± 0.05 ^b^	9.88 ± 0.08 ^b^
Growth in milk^1^			
0 h	-	6.46 ± 0.04 ^d^	5.83 ± 0.09 ^b^
24 h	-	8.62 ± 0.11 ^c^	7.47 ± 0.05 ^a^
Refrigerated storage^1^			
14 d	-	9.07 ± 0.05 ^a^	7.11 ± 0.38 ^a^
21 d	-	8.88 ± 0.02 ^b^	7.51 ± 0.05 ^a^
28 d	-	9.03 ± 0.07 ^a^	7.56 ± 0.01 ^a^
OPA (A_340_)^2^	0.163 ± 0.002 ^b^	0.196 ± 0.004 ^a^	0.165 ± 0.001 ^b^
Milk pH^2^	6.236 ± 0.002 ^a^	5.033 ± 0.029 ^b^	6.265 ± 0.010 ^a^

^1^Microbial levels were expressed as log CFU/mL. Values were expressed as mean ± standard deviation of duplicate determinations in two experiments (*n* = 4). Means within the same column with different superscripts differ significantly at *P* < 0.01

^2^Values were expressed as mean ± standard deviation of duplicate determinations in two experiments (*n* = 4). Means within the same row with different superscripts differ significantly at *P* < 0.01

When growing in milk, *L. paracasei* INIA P708 increased its level more than 2 log units after 24 h incubation (Table 3). The increase for *L. rhamnosus* GG only reached 1.6 log units. During refrigerated storage, the INIA P708 strain still increased its levels, whereas the GG strain was only able to maintain them.

The INIA P708 strain also showed proteolytic and acidifying activities (Table 3). The values recorded for the GG strain did not significantly (*P* > 0.01) differ from non-inoculated milk. Although pH values in INIA P708 milk cultures did not reach the level to precipitate caseins (pH = 4.6), EPS production turned them into a solid state. On the contrary, GG milk cultures remained in a liquid state.

Salt had a significant (*P* < 0.01) effect on the growth of both strains (Table 4). INIA P708 was more sensitive to salt than GG. When INIA P708 was inoculated at 7 log CFU/mL, increasing salt concentrations resulted in A_600_ reductions up to 30%. At 5 log CFU/mL, a 30% A_600_ reduction was already observed for the 3% NaCl culture, and hardly any growth was recorded for the 4 and 5% NaCl cultures. However, for GG, we only observed up to 15% reductions at the highest salt concentration, irrespective of the inoculation level.

**Table 4:** Absorbance at 600 nm after incubation (at 37 °C for 24 h under anaerobic conditions) in MRS with different NaCl concentrations.

NaCl (%)	Strains (inocula)			
	INIA P708 (7 log CFU/mL)	GG (7 log CFU/mL)	INIA P708 (5 log CFU/mL)	GG (5 log CFU/mL)
0	2.32 ± 0.01 ^a^	2.16 ± 0.03 ^ab^	2.18 ± 0.01 ^a^	2.13 ± 0.02 ^b^
1	2.27 ± 0.01 ^b^	2.21 ± 0.03 ^a^	2.10 ± 0.01 ^b^	2.20 ± 0.02 ^a^
2	2.18 ± 0.00 ^c^	2.20 ± 0.03 ^a^	1.96 ± 0.02 ^c^	2.11 ± 0.02 ^b^
3	2.10 ± 0.02 ^d^	2.11 ± 0.02 ^b^	1.52 ± 0.02 ^d^	2.03 ± 0.02 ^c^
4	1.92 ± 0.01 ^e^	1.97 ± 0.03 ^c^	0.05 ± 0.01 ^e^	1.94 ± 0.03 ^d^
5	1.64 ± 0.02 ^f^	1.87 ± 0.00 ^d^	0.01 ± 0.00 ^f^	1.79 ± 0.02 ^e^

Values were expressed as mean ± standard deviation of duplicate determinations in three experiments (*n* = 6). Means within the same column with different superscripts differ significantly at *P* < 0.01

For both strains, microbial levels in milk supplemented with lactulose, FOS, or GOS were not significantly (*P* > 0.01) different from levels in control milk (Table 5). However, significantly (*P* < 0.01) lower pH values were recorded in cultures with FOS or GOS for both strains.

**Table 5:** Microbial levels and pH values after growth in milk (at 37 °C for 24 h under anaerobic conditions) with several prebiotic ^1^.

Parameter	Strains	
	INIA P708	GG
Microbial levels^2^ 0 h		
Control milk	5.72 ± 0.05 ^a^	5.63 ± 0.06 ^a^
Milk + Lu	5.68 ± 0.06 ^a^	5.55 ± 0.10 ^a^
Milk + FOS	5.70 ± 0.03 ^a^	5.54 ± 0.12 ^a^
Milk + GOS	5.69 ± 0.03 ^a^	5.58 ± 0.14 ^a^
Microbial levels 24 h		
Control milk	8.86 ± 0.07 ^a^	8.32 ± 0.07 ^a^
Milk + Lu	8.86 ± 0.15 ^a^	8.33 ± 0.07 ^a^
Milk + FOS	8.94 ± 0.08 ^a^	8.39 ± 0.09 ^a^
Milk + GOS	9.00 ± 0.10 ^a^	8.41 ± 0.06 ^a^
pH 0 h		
Control milk	6.79 ± 0.04 ^a^	6.79 ± 0.04 ^a^
Milk + Lu	6.83 ± 0.01 ^a^	6.83 ± 0.01 ^a^
Milk + FOS	6.85 ± 0.02 ^a^	6.85 ± 0.02 ^a^
Milk + GOS	6.79 ± 0.02 ^a^	6.79 ± 0.02 ^a^
pH 24 h		
Control milk	6.02 ± 0.06 ^a^	6.51 ± 0.02 ^a^
Milk + Lu	5.97 ± 0.04 ^a^	6.51 ± 0.01 ^a^
Milk + FOS	5.89 ± 0.02 ^b^	5.84 ± 0.04 ^c^
Milk + GOS	5.82 ± 0.02 ^b^	6.15 ± 0.02 ^b^

^1^Prebiotic compounds: *Lu* lactulose, *FOS* fructooligosaccharides, and *GOS* galactooligosaccharides

^2^Microbial levels were expressed as log CFU/mL. Values were expressed as mean ± standard deviation of duplicate determinations in three experiments (*n* = 6). Means within the same column with different superscripts differ significantly at *P* < 0.01

### Safety evaluation

Antibiotic resistance and toxicity are important safety aspects to be evaluated for potential probiotic strains (Rodríguez et al. 2012). Concerning antibiotic resistance, both strains were susceptible to chloramphenicol, erythromycin, rifampicin, and tetracycline and resistant to kanamycin and vancomycin.

Neither the INIA P708 strain nor the GG strain was able to produce biogenic amines from histidine or tyrosine.

The ability to degrade mucin is considered one of the valuable indicators of potential pathogenicity and local toxicity of intestinal bacteria. No mucinolytic activity was found in either INIA P708 or GG strains.

### Sequencing and general genomic features

The probiotic traits and technological properties indicate that INIA P708 can be of potential interest for food applications. Since intentional addition of microorganisms to the food chain includes an obligatory genomic analysis (European Food Safety 2024), the entire INIA P708 genome was sequenced and analyzed.

A total of 2 × 1,353,386 Illumina paired-end reads and 37,025 PacBio HiFi reads were obtained. After trimming and decontamination, a total of 1,551,739 short reads and 37,024 long reads remained, resulting in a hybrid assembly with a genomic size of 3,143,551 bp (N50 of 3,076,756) and a GC content of 46.28%. INIA P708 genome was composed of a circular chromosome (3,076,756 bp) and one plasmid (66,795 bp). It was deposited in Genbank (BioProject ID PRJNA 1401546, BioSample accession SAMN54566742). This fully resolved genome had a high average coverage depth (152x), and a predicted completeness (99.89%), with a very low (0.37%) possible contamination.

### Species confirmation

Taxonomy based on ANI revealed that the genome of strain INIA P708 was closely related to the *L. paracasei* strains VHProbi F22 (GCF_022478075.1) and *L. paracasei* subsp. *paracasei* 01 (GCF_024665815.1), with ANI values above 99.996% in both cases. Additionally, the taxonomic analysis performed using the TYGS web server indicated that strain INIA P708 showed similarity to *L. paracasei* JCM 8130 (dDDH = 87.6%), *L. paracasei* ATCC 25302 (dDDH = 87.5%), and *L. paracasei* subsp. *tolerans* DSM 20258 (dDDH = 85.2%). Therefore, the results obtained using both methodologies confirmed that INIA P708 belongs to the species *Lacticaseibacillus paracasei*.

### Genomic annotation

Structural annotation performed by Bakta revealed a total of 3168 genes, distributed as 3075 protein-coding sequences, 8 sORFs, 61 tRNA genes, 15 rRNA genes, 1 tmRNA, and 8 ncRNA. Among the predicted CDS, only 193 genes were annotated as hypothetical proteins with unknown function.

**Fig. 2:**
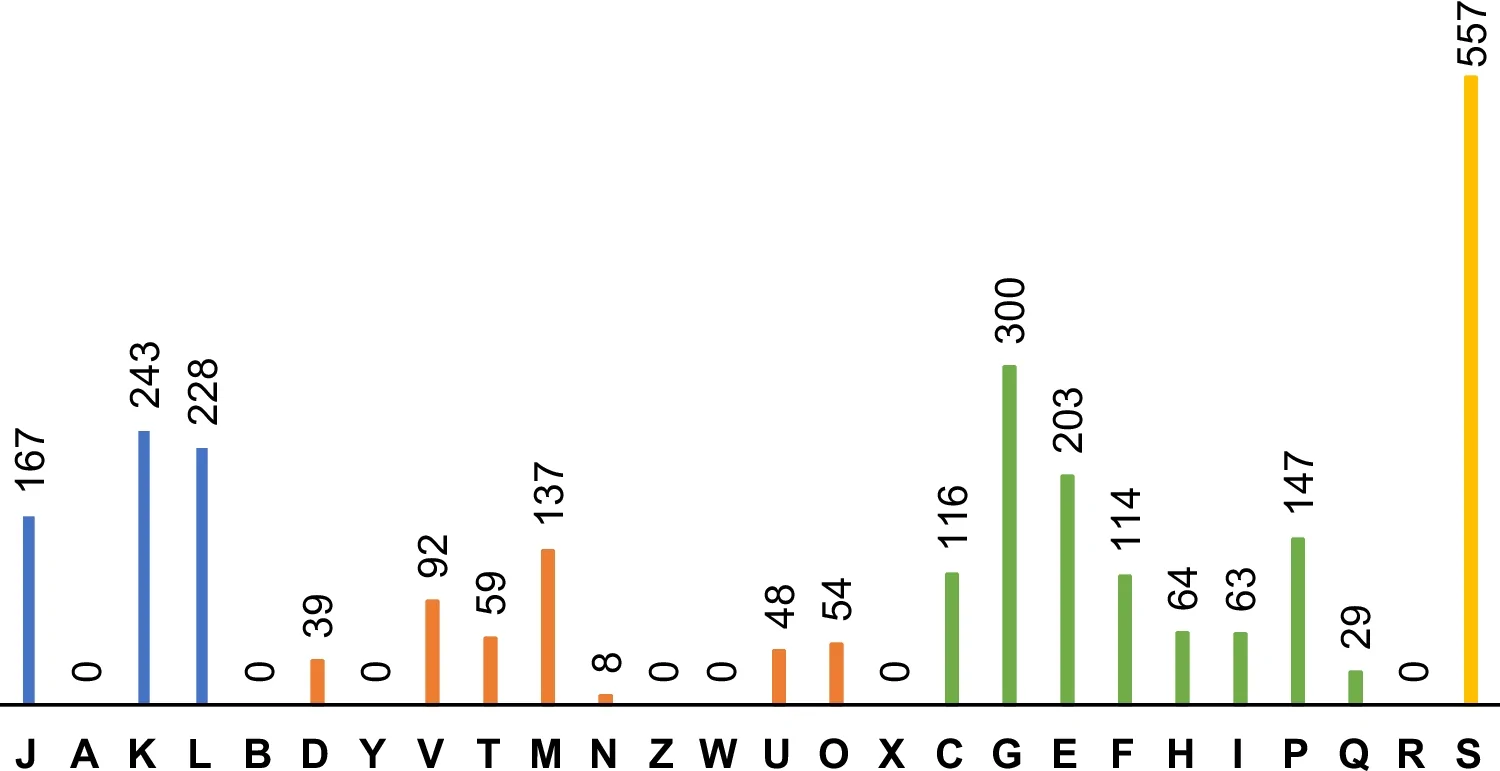
COG category distribution of INIA P708 functional annotation results. COG one letter code descriptions: (1) information storage and processing (blue bars): [J] translation, ribosomal structure and biogenesis; [A] RNA processing and modification; [K] transcription; [L] replication, recombination and repair; [B] chromatin structure and dynamics. (2) Cellular processes and signaling (orange bars): [D] cell cycle control, cell division, chromosome partitioning; [Y] nuclear structure; [V] defense mechanisms; [T] signal transduction mechanisms; [M] cell wall/membrane/envelope biogenesis; [N] cell motility; [Z] cytoskeleton; [W] extracellular structures; [U] intracellular trafficking, secretion, and vesicular transport; [O] posttranslational modification, protein turnover, chaperones. (3) Metabolism (green bars): [C] energy production and conversion; [G] carbohydrate transport and metabolism; [E] amino acid transport and metabolism; [F] nucleotide transport and metabolism; [H] coenzyme transport and metabolism; [I] lipid transport and metabolism; [P] inorganic ion transport and metabolism; [Q] secondary metabolites biosynthesis, transport and catabolism. (4) Poorly characterized (yellow bars): [R] general function prediction only; [S] function unknown.

Out of the 3083 protein sequences annotated by Bakta (3075 CDS and 8 sORFs), eggNOG-mapper was able to functionally annotate 2832 sequences. Of these, 2521 were assigned to COG families comprising 19 functional categories, while the remaining sequences were functionally unknown (Fig. 2). The most abundant categories were carbohydrate transport and metabolism (G: 300), transcription (K: 243), and replication, recombination, and repair (L: 228). Regarding KEGG terms (Table S1), 1691 protein sequences (59.71% of the functionally annotated sequences by eggNOG-mapper) were assigned to 16 functional categories related to metabolic pathways. Among these, most were related to carbohydrate metabolism (252), environmental information processing (223), and genetic information processing (167). KEGG pathway reconstructions predicted complete metabolic routes for glycolysis, the tricarboxylic acid (TCA) cycle, amino acid biosynthesis, and fatty acid biosynthesis, supporting a potentially robust central metabolic network.

Carbohydrate-active enzymes (CAZymes) are produced by the microbiome in the human intestinal gut and play a key role in the synthesis and degradation of complex polysaccharides and their derivatives (Kaoutari et al. 2013). According to eggNOG-mapper results, INIA P708 harbors 42 genes classified under 5 CAZy families (Table S2). The protein sequences annotated by CAZy were dominated by glycosyltransferases (GT, *n* = 22) and glycoside hydrolases (GH, *n* = 16). The carbohydrate esterase and polysaccharide lyase families were each represented by one sequence, whereas the carbohydrate-binding module family included two proteins, also classified as glycoside hydrolases.

There is genomic evidence of stress resilience, metabolic versatility, and host adaptation supporting the probiotic potential of *L. paracasei* INIA P708. A list of stressor/probiotic marker genes related to resistance to different (thermal, acidic, bile, osmotic, oxidative, and metal) stressors, adhesion ability, gut persistence, and immunomodulation was previously suggested (Kandasamy et al. 2024). The genes identified in the INIA P708 strain (Table S3) support its probiotic and technological potential. The genes related to temperature and osmotic stress (e.g., *dnaK*, *cspC*, *osmF*, *glpF*) suggest resilience during food processing and storage, while those involved in acid and bile resistance (*atpA*, *pyk*, *oppA*, *cbh*) support survival through the GIT harsh conditions. Furthermore, genes involved in adhesion and gut persistence (*lspA*, *eno*, *celA*, *celB*) may facilitate colonization and interaction with the host epithelium and adaptation to the intestinal environment. In addition, comparison of the INIA P708 strain with *L. plantarum* WCFS1 strain, known to induce IL-10 and IL-12 secretion by dendritic cells (Meijerink et al. 2010), showed that several genes associated with immunomodulatory capacity are highly conserved. These include genes related to the bacteriocin export system (*plnG*, *abpD*), the phosphotransferase system (PTS EIIA and EIIC), and key transcriptional regulators (*gntR*, *xre*).

### Mobile genetic elements and defense systems

#### Plasmids

The assembly contained two contigs: the chromosome and one plasmid (pINIA_P708). This plasmid was identified using PlasmidFinder and geNomad. Spanning 66,795 bp, it harbors a total of 74 genes, including the MOBP1 and FATA_prgIb markers. According to PlasmidFinder, the plasmid carried a RepA_N-type replicon with 85.52% identity and 49.39% coverage compared to plasmid 2 of *L. paracasei* subsp. *paracasei* 8700:2 (CP002393.1). It also encoded a site-specific recombinase (DNA invertase), a type I restriction-modification system, enzymes involved in sugar utilization (a lactose-specific PTS system, a 6-phospho-beta-galactosidase, a glycosyltransferase), and in flavor formation (a cystathionine beta-synthase, a cysthathionine gamma-lyase, and a serine acetyltransferase), an ATP-utilizing enzyme of the PP-loop superfamily, ten repeat regions, six transposases, and 44 hypothetical proteins. This plasmid was identical to the one of *L. paracasei* VHProbi F22 (GCF_022478075.1), the strain with which INIA P708 exhibited the highest ANI.

#### Temperate bacteriophage prediction

A total of 5 high confidence predicted prophage regions (with medium or higher quality) were identified (Table 6). Three of them showed high coverage against *Lactobacillus* phages iA2, PLE2, and PLE3, respectively. Although *Lacticaseibacillus* phage C4.1 was only covered at 16%, we considered it as a putative prophage because the CheckV predicted integrity is over 50%. Lastly, an uncharacterized *Caudoviricetes* sp. isolate was also recovered.

**Table 6:** List of phages annotated in the *L. paracasei* INIA P708 genome.

Region	Length	Id^1^	Score	Quality	Integrity	Best BLAST result	Coverage
1	24.67	P	80/150	Medium	54.82	*Lacticaseibacillus* phage C4.1 (OP807341.1)	16
2	42.57	P	150/150	High	95.54	*Lactobacillus* phage iA2 (KR905068.1)	80
2	33.77	geN	0.9983/1	High	97.97	*Lactobacillus* phage iA2 (KR905068.1)	100
3	39.68	P	150/150	Medium	88.37	*Lactobacillus* phage PLE2 (KU848187.1)	88
3	35.68	geN	0.9987/1	Medium	88.91	*Lactobacillus* phage PLE2 (KU848187.1)	97
4	46.05	P	150/150	High	100	*Lactobacillus* phage PLE3 (KU848186.1)	88
4	44.05	geN	0.9987/1	High	100	*Lactobacillus* phage PLE3 (KU848186.1)	92
5	13.67	P	90/150	High	96.72	*Caudoviricetes* sp. isolate ctG1U4 (BK021574.1)	100
5	13.50	geN	0.9982/1	High	95.63	*Caudoviricetes* sp. isolate ctG1U4 (BK021574.1)	100

^1^*Id* identification method: *P* PHASTEST, *geN* geNomad, *Integrity* CheckV integrity

#### Defense systems

Analysis of CRISPR/Cas systems revealed a complete subtype II-A system in the bacterial chromosome. The Cas operon consisted of the genes csn2, cas2, cas1, and cas9, while the CRISPR cluster included 20 repeats and spacers. In addition to this system, PADLOC identified several defense systems in the bacterial chromosome, including AbiD, AbiO-Nhi family, DMS_other, HEC-06, PD-Lambda-1, and PDC-S50. Moreover, a type I restriction-modification system was detected in the plasmid.

### Lack of potentially adverse genes

None of the executed programs was able to identify AMR genes in *L. paracasei* INIA P708, indicating that this strain can be considered safe regarding the potential dissemination of antimicrobial resistance genes. Similarly, no virulence factors were detected using VirulenceFinder or VFDB. Additionally, WSPC reported a 1% probability of INIA P708 being pathogenic, while the probability reached 19.8% with PathogenFinder. However, both values were very low, and neither of the methods identified INIA P708 as a pathogenic strain.

### *In silico* analysis of antimicrobial potential

**Fig. 3:**
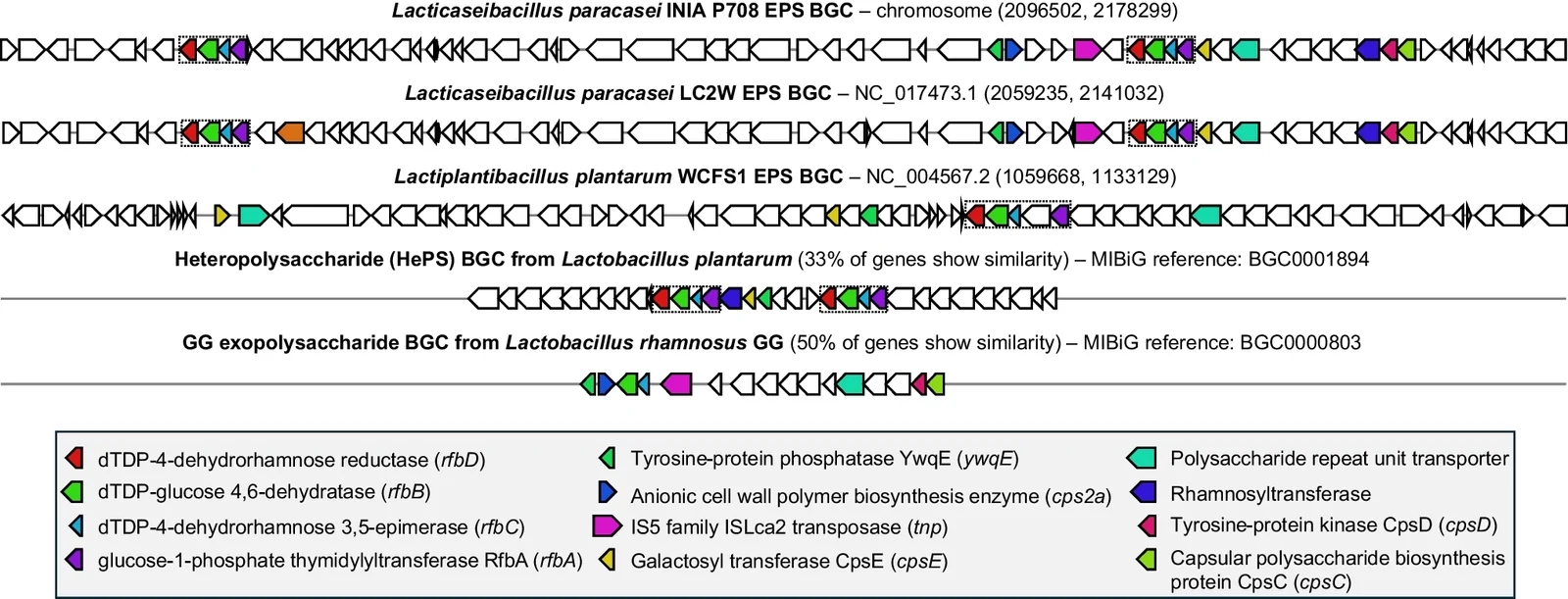
Comparative analysis of the exopolysaccharide (EPS) biosynthetic gene cluster from strain INIA P708 and related polysaccharide clusters. The analysis was performed using the KnownClusterBlast module within antiSMASH, and the resulting figure was modified to improve interpretability. Homologous clusters were retrieved from the MIBiG 3.1 database. Conserved genes shared among clusters are represented using consistent color coding reflected in the legend. The *rfb*DBCA gene clusters are marked with a dotted rectangle.

The biosynthetic gene clusters (BGCs) in *L. paracasei* INIA P708 may have the ability to produce bacteriocins. Results from antiSMASH revealed 11 potential BGCs, classified as saccharide (8 + 1 in the plasmid), fatty acid (1), and RiPP-like (1) types. The saccharide clusters were detected with loose strictness, suggesting they may be poorly defined or related to primary metabolites. Nevertheless, two of the potential BGCs identified in the bacterial chromosome contained genes present in the molybdenum cofactor biosynthetic gene cluster from *Staphylococcus carnosus* (Neubauer et al. 1998) and the heteropolysaccharide (HePS) biosynthetic gene cluster from *Lactiplantibacillus plantarum* (Prechtl et al. 2018). The first cluster is essential for molybdenum cofactor biosynthesis, which is crucial for nitrate reductase activity in *S. carnosus*. The second cluster produces a heteropolysaccharide associated with the structure and function of the bacterial cell wall (Fig. 3), which could correspond to the experimentally identified EPS. This cluster is very similar to that of *L. casei* LC2W (Chen et al. 2011) and displays an organization consistent with complex heteropolysaccharide-producing systems, including genes involved in precursor biosynthesis as well as in polysaccharide chain assembly and modification. In particular, the complete dTDP-rhamnose biosynthetic pathway is present, comprising *rfb*A (INIAP708_10795), *rfb*B (INIAP708_10785), *rfb*C (INIAP708_10790), and *rfb*D (INIAP708_10780), indicating that this strain has the genetic capacity to incorporate rhamnose into the EPS structure (Zhang et al. 2023). In addition to precursor synthesis, the cluster contains diverse glycosyltransferases involved in assembly of the polysaccharide repeating unit and enzymes responsible for polysaccharide modification. In the LC2W EPS, too, acetylated sugars were identified (Ai et al. 2016), as were visualized by WGA labelling in our samples. EPS synthesis in *L. paracasei* INIA P708 thus seems to follow the same general system as described for other bacteria in this family.

**Fig. 4:**
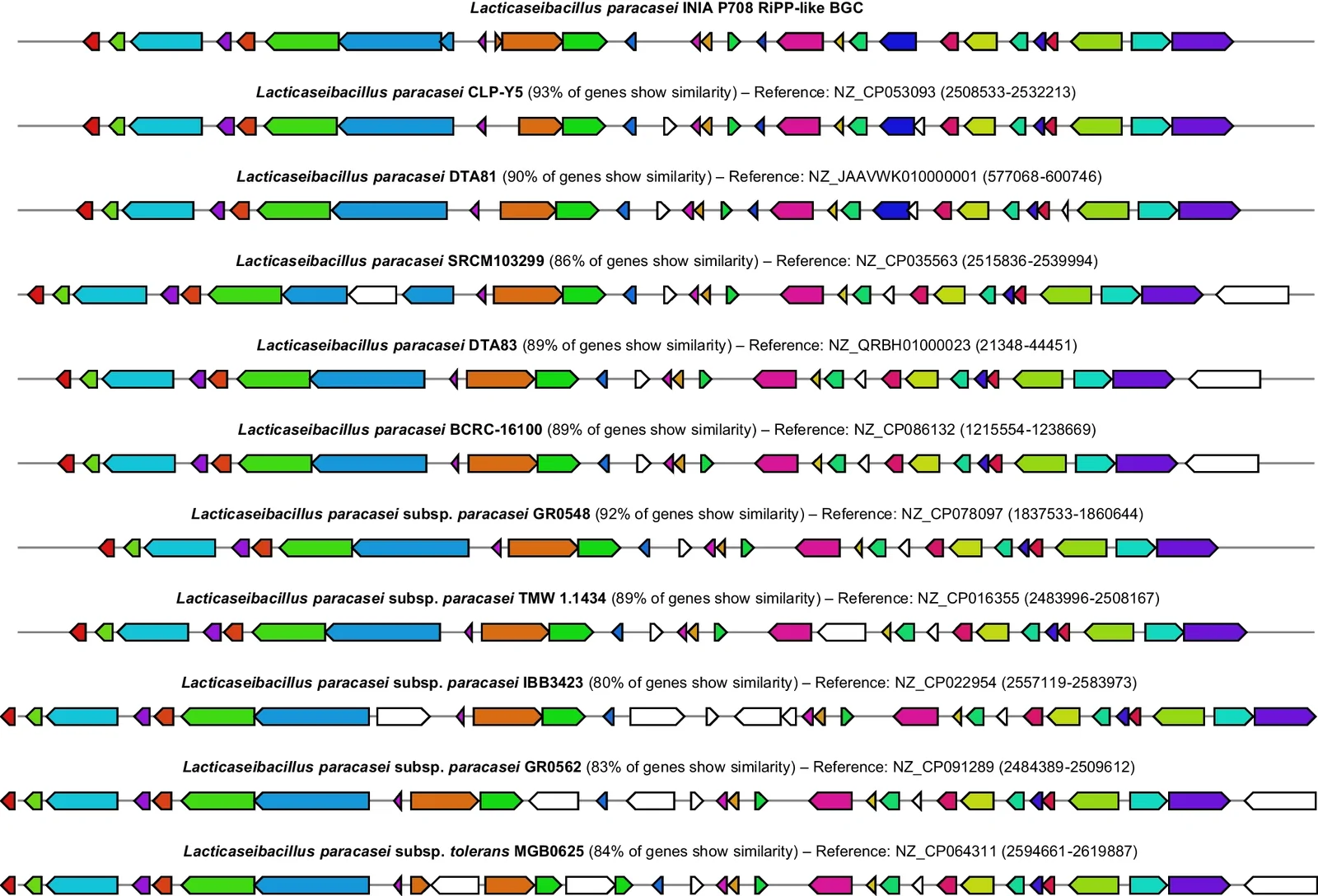
Comparative analysis of the RiPP-like biosynthetic gene cluster from strain INIA P708 and related clusters. The analysis was performed using the ClusterBlast module within antiSMASH, and the resulting figure was modified to improve interpretability. Homologous clusters were retrieved from antiSMASH database. Conserved genes shared among clusters are represented using consistent color coding.

The only cluster identified with higher detection strictness was the RiPP-like type, which contains 10 genes associated with bacteriocin synthesis (Fig. 4). Although this cluster has been previously detected, its specific function has not been studied (Lemos Junior et al. 2019; Tarrah et al. 2021).

BAGEL4 identified three potential BGCs encoding enterolysin A (a class III bacteriocin) and LSEI 2163 and LSEI 2386 (both class II bacteriocins). Furthermore, within the same LSEI 2386 cluster, two sequences of the β-chain of enterocin X and one of carnocin CP52 were identified, among other class II bacteriocins. Additionally, the overlap of this cluster with the RiPP-like cluster identified by antiSMASH strengthens the hypothesis that this cluster confers *L. paracasei* INIA P708 diverse antibacterial functions.

## Discussion

In this work, on top of showing the probiotic traits and technological properties of the *L. paracasei* INIA P708 strain, we successfully resolved its chromosome and identified and characterized one plasmid. We also focused on the annotation of antimicrobial resistance genes and biosynthetic gene clusters for probiotic and other secondary metabolites biosynthesis.

Although members of the genus *Lacticaseibacillus* have genome sizes ranging from 1.93 to 3.14 Mbp, and G + C contents between 46 and 58.0% (Zheng et al. 2020), the *L. paracasei* type strain (DSM 5622 = ATCC 25302 = LMG 13087) has a genome size of the 2.88 Mbp and a 46.5% GC content (Zheng et al. 2020). The genomic size of *L. paracasei* INIA P708 (3.14 Mbp) was bigger, and its G + C content (46.3%) slightly lower than the ones of the type strain. The INIA P708 chromosome size (3.08 Mbp) was closer to the 3.06 Mbp of the *L. paracasei* INIA P272 draft genome (Rodrigo-Torres et al. 2022), another strain of human origin from the INIA collection. Both INIA strains had an identical GC content. The chromosome size and GC content of strain INIA P708 were identical to the ones of *L. paracasei* VHProbi F22 (Cui et al. 2022), a commercial probiotic strain originally isolated from Mongolian cheese, and the strain with which INIA P708 exhibited the highest ANI.

Plasmids larger than 20 Kbp are frequently found in *L. paracasei* strains (Smokvina et al. 2013). They have been shown to encode many unique functions as EPS biosynthesis, bacteriocin biosynthesis, proteolysis and flavor formation, lactose metabolism, or heavy metal resistance, among others (Smokvina et al 2013). Indeed, the *L. paracasei* INIA P708 plasmid (66.8 Kbp) encodes enzymes involved in sugar utilization and in flavor formation. Similar plasmid encoded functions were already reported in plasmids present in four out of five reference strains and 31 out of 34 *L. paracasei* strains studied by Smokvina et al. (2013). The INIA P708 plasmid is identical to the one of *L. paracasei* VHProbi F22 (Cui et al. 2022). The presence of enzymes involved in flavor formation also supports the fact that fermented milk products elaborated with the INIA P708 strain were described as sweet, aromatic, and pleasant in sensory tests (Rodríguez-Mínguez et al. 2024). Similar descriptors were attributed to fermented milk products with the GG strain (Rodríguez-Mínguez et al. 2024).

Using genus and species-specific primers directed to variable regions of the 16S rRNA gene (Rodríguez et al. 2012), the INIA P708 strain was previously identified as *Lacticaseibacillus paracasei*. In this work, both the taxonomy based on ANI and the taxonomic analysis performed using the TYGS web server confirmed that INIA P708 belongs to this species.

Although ANI values of the INIA P708 strain with the VHProbi F22 and *L. paracasei* subsp. *paracasei* 01 strains were above 99.996%, the genome of strain INIA P708 was annotated as containing 3075 protein-coding sequences, 8 sORFs, 61 tRNA genes, 15 rRNA genes, 1 tmRNA, and 8 ncRNA. Among the predicted CDS, 193 genes were annotated as hypothetical proteins with unknown function. Comparisons with other *L. paracasei* strains are to be taken with caution, due to the use of different annotation methods. For example, the genome of VHProbi F2 contained 2930 protein-coding genes, 61tRNA genes, 15 rRNA genes, 3 ncRNA genes, and 81 pseudogenes (Cui et al 2022). Similarly, *L. paracasei* subsp. *paracasei* 01, a Chr. Hansen probiotic strain, contained 2938 protein-coding genes, 61 tRNA genes, 15 rRNA genes, 3 ncRNA, and 97 pseudogenes.

A complete CRISPR/Cas subtype II-A system and several defense systems (including AbiD, AbiO-Nhi family, DMS_other, HEC-06, PD-Lambda-1 and PDC-S50) were located in the INIA P708 chromosome. This CRISPR/Cas system provides immunity against foreign DNA (Makarova et al. 2020). The other systems provide a variety of protective mechanisms against foreign phage infections and other mobile genetic elements, which might favor the genome stability. Moreover, a type I restriction-modification system was detected in the plasmid, which also contributes to bacterial defense by recognizing and cleaving foreign DNA (De Ste Croix et al. 2017).

Regarding the ropy phenotype and EPS production, EPS represent a large group of carbohydrate polymers, of interest for health- and food-applications (Llamas-Arriba et al. 2019). They are produced by a variety of bacteria and can be either tightly cell-associated, loosely attached to the surface, or secreted into the extracellular environment (Mozzi et al. 2006). The binding of WGA to cell surface structures has been reported to be characteristics of Gram-positive bacteria, where peptidoglycan-containing cell walls are directly exposed to the external medium and easily accessible for lectins (Strathmann et al. 2002). Thus, a proportion of the WGA could be bound by the peptidoglycan of both strains, as was observed in the CSLM micrographs. Others have observed that the GG cell surface contains two major types of polysaccharides: a long galactose-rich EPS with small amounts of L-rhamnose and N-acetyl-D-glucosamine residues and a short glucose/mannose-rich polysaccharide (Francius et al. 2009). Labelling GG with two different lectins produced distinct patterns in our experiments, one associated with the cell surface and another disperse in the medium. While WGA detected the cell-associated N-acetyl-D-glucosamine in the long EPS, the scattered signal observed for GG probably was due to the binding of ConA to the short glucose/mannose-rich EPS (Francius et al. 2009). For INIA P708, both lectins produced cell-associated patterns, although we observed that some cells were not labelled with ConA. As a small amount of ConA label was found in the growth medium of INIA P708, this strain also seems to shed part of its glucose/mannose-rich EPS.

The INIA P708 EPS has not been characterized yet at the molecular level. The heteropolysaccharide (HePS) coding region found by antiSMASH, and located in the chromosome, is a large region (81.8 kb and 71 CDS) containing some gene duplications and subclusters similar to the HePS cluster of *L. casei* LC2W (Chen et al. 2011) and the GG EPS cluster (Fig. 3). As in other EPS clusters, these genes are organized in specific regions involved in regulation, chain length determination, biosynthesis of the repeat unit, polymerization, and export (Dabour and LaPointe 2005). The glycosyltransferase genes in the cluster suggested that the EPS may be composed of galactose, glucose, and rhamnose for the heteropolysaccharide repeating unit with partial acetyl substitution. The presence of glucose residues in its composition supports the union of ConA to the INIA P708 EPS.

Importantly, this predicted monosaccharide composition may also provide clues about its potential biological activity, since the structural features of LAB-derived EPSs are closely linked to their functional, immunomodulatory, and anti-inflamatory properties. Several studies conducted before indicate that EPS derived from ropy LAB has stronger anti-inflammatory properties as compared to non-ropy LAB (Nikolic et al. 2012). Also rhamnose and other monosaccharides, recurrent constituents of heteropolysaccharides produced by lactic acid bacteria, are widely recognized as important determinants of EPS bioactivity and immunomodulatory potential (van Calsteren et al. 2002; Fuso et al. 2023; Zhang et al. 2023). In this context, LAB-derived EPS have been reported to interact with pattern-recognition receptor pathways, including TLR2/TLR4-associated signaling, and to promote anti-inflammatory responses characterized by increased IL-10 and reduced pro-inflammatory cytokines (Laiño et al. 2016), which might have synergistic effect with the presence of other conserved immunomodulatory genes (Table S3).

Concerning probiotic traits, short-chain fatty acids play an important role in health maintenance (Rodrigo-Torres et al. 2022). Acetate kinase (*ackA*), a common gene for acetate biosynthesis, is present in the INIA P708 genome. Seven copies of D- and L-lactate dehydrogenase genes and three predicted L-forms were also present in the INIA P708 chromosome. However, we did not find the phosphotransacetylase (*pta*) gene, genes for butyrate biosynthesis as butyrate kinase (*buk*) or phosphate butyryl transferase (*ptb*), or other genes also contributing to SCFA biosynthesis, like thioesterases (Zhao et al. 2019). On the contrary, *L. paracasei* INIA P272 encoded genes for butyrate production (*buk* and *ptb*) and homologs to *E. coli* thioesterases for acetate synthesis (acyl-CoA thioester hydrolase (*ybhC*), acyl-CoA-thioestherase (*yciA*), and thioesterase 1 (*tesA*)), but not 1,4-dihydroxy-2-naphthoyl-CoA hydrolase (*menI*) (Rodrigo-Torres et al. 2022).

Many lactobacilli have the ability to synthesize vitamins, specially of the B group, even though this ability is strain-specific (Teran et al. 2021). All genes of the thiamine (vitamin B1) biosynthesis pathway (*thiDEM* genes) are present in the INIA P708 genome. This fact confers another potential probiotic value to this strain. Similarly, this complete biosynthetic pathway is also present in strain INIA P272 (Rodrigo-Torres et al. 2022).

The GG strain possesses a high biofilm forming capacity and was used as positive control to develop the biofilm staining method employed in this work (Lee et al. 2019). The major adhesion mechanism in *L. rhamnosus* GG is the *spaCBA* pili fiber system operon (Spacova et al. 2020). The pilus backbone is formed by SpaA pilin subunits, covalently linked to each other by the transpeptidase activity of a pilin-specific sortase. SpaC pilin subunits, with adhesive function, are located at the pilus tip and on the pilus fibers. At the pilus base, one SpaB pilin subunit anchors it to the peptidoglycan layer, and a few SpaB subunits are on the pilus fibers. The genes coding for the three pilins subunits and the pilin-specific sortase are usually localized at the same locus (Mandlik et al. 2008). Two pilus clusters, each with a pilin-specific sortase, are present in the INIA P708 chromosome. Although we did not observe biofilm formation under the tested conditions, we cannot rule out that these pili are produced in the intestinal tract under *in vivo* conditions in a similar way to the Tad pilus genes of the *B. breve* UCC 2003 strain (O'Connell Motherway et al. 2011).

The antimicrobial activity observed against the Gram-positive *Listeria monocytogenes* and against Gram-negative microorganisms as *Klebsiella oxytoca* and *Salmonella enterica* could be related to the presence of several biosynthetic gene clusters detected by antiSMASH and BAGEL4. The RiPP-like cluster identified by antiSMASH (Fig. 4) has also been found in two other *L. paracasei* strains (Lemos Junior et al. 2019; Tarrah et al. 2021), but its specific function has not been studied. BAGEL4 identified three potential BGCs encoding enterolysin A, LSEI 2163, and LSEI 2386. Enterolysin A is a thermostable class III bacteriocin that degrades cell walls and exhibits activity against Gram-positive bacteria. LSEI 2163 and LSEI 2386 are class II bacteriocins that demonstrated antimicrobial activity against some lactobacilli and *Listeria* species (Kuo et al. 2013). Furthermore, within the same LSEI 2386 cluster, two sequences of the β-chain of enterocin X and one of carnocin CP52 were identified, among other class II bacteriocins. Enterocin X is a bacteriocin composed of two antibacterial peptides that display synergistic antibacterial activity (Hu et al. 2010). Carnocin CP52 is a bacteriocin with anti-Listeria activity (Herbin et al. 1997). Additionally, the overlap of this cluster with the RiPP-like cluster identified by antiSMASH strengthens the hypothesis that this cluster confers *L. paracasei* INIA P708 diverse antibacterial functions. Similarly, enterolisin A and carnosin CP52 bacteriocins, and three other possible BGCs were detected in strain INIA P272 (Rodrigo-Torres et al. 2022). Further *in vitro* assays are needed to unveil if INIA P708 produces bacteriocins.

Considering technological properties, we observed that INIA P708 is able to grow in milk, increasing overall proteolysis and decreasing pH. INIA P708 possesses a PII-type proteinase, several aminopeptidases, two lactose-specific PTS systems (one in the plasmid and another one in the chromosome), and several galactosidases. In contrast, the GG strain is neither able to metabolize lactose nor to degrade milk caseins (Hussain et al. 2021).

When subjected to osmotic stress, bacterial cells undergo significant physiological changes including the induction of stress proteins as well as the accumulation of compatible solutes such as betaine, carnitine, and trehalose (Prasad et al. 2003). We showed that, in spite of the osmotic stress mechanisms present in INIA P708 chromosome, INIA P708 viability was more compromised than that of GG. In a similar study, including three probiotic bacterial strains (one *L. casei*, one *L. acidophilus*, and one *B. longum* strains), the *L. casei* strain was the least affected by high salt concentrations (Gandhi and Shah 2015).

When growing in milk with several prebiotic sugars, we recorded no significant (*P* > 0.01) growth differences for INIA P708 or GG with respect to growth in plain milk. Similar results were recorded for the *L. paracasei* INIA P272 and *L. rhamnosu*s INIA P344 strains in milk supplemented with FOS or GOS with respect to control milk (Langa et al. 2019). However, growth in milk with FOS or GOS resulted in significantly (*P* < 0.01) lower pH values for INIA P708 and GG. On the contrary, no effect of FOS or GOS on milk pH was recorded for *L. paracasei* INIA P272 or *L. rhamnosus* INIA P344 (Langa et al. 2019).

Regarding safety traits, we observed that both the INIA P708 and the GG strains were resistant to kanamycin and vancomycin. However, none of the analyses performed with ResFinder v4.4.2, AMRFinderPlus v3.12.8, and RGI-CARD v6.0.3 was able to identify antimicrobial resistance (AMR) genes in *L. paracasei* INIA P708 genome. Those resistances must be due to intrinsic mechanisms, and they might be of interest in therapies for antimicrobial-associated diarrhea (Rodríguez et al. 2012). Kanamycin resistance has also been reported for several lactobacilli that did not possess AMR genes (Rodrigo-Torres et al. 2022; Rozman et al. 2023). In conclusion, the INIA P708 strain can be considered safe regarding the potential dissemination of antimicrobial resistance genes.

Biogenic amines are primarily produced by microbial decarboxylation of free amino acids. High concentrations can lead to toxicological effects such as headaches, nausea, and hypertension, but trace amounts are essential for normal metabolic and physiological functions. Among the biological functions are neuromodulation, maintenance of the gut health and motility, gastric acid secretion, regulation of immune response, cell growth, and gene expression (Sandoval and Shah 2025). Lactic acid bacteria can either produce or degrade biogenic amines into non-toxic compounds, using substrate-specific decarboxylases or amine-degrading enzymes (such as amine oxidase and multicopper oxidase), respectively (Banicod et al. 2025). Under the tested conditions, no biogenic amines were produced by INIA P708. However, two Orn/Lys/Arg decarboxylases, one aspartate 4-decarboxylase (*aspD*), and one multicopper oxidase (*sufl*) genes were found among the annotated genes. Therefore, it is possible that INIA P708 is able to produce biogenic amines and degrade them. Bioinformatic analysis of human gut bacterial genomes revealed that Arg decarboxylases were the most common decarboxylases in these bacteria and played a crucial role in microbial survival under extreme acidic conditions (Sandoval and Shah 2025). Aspartate 4-decarboxylase, that decarboxylates aspartate into L-alanine, was also found in the genomes of other *L. paracasei* strains (Sandoval and Shah 2025).

INIA P708 did not degrade mucine. Similarly, no mucinolytic activity was found in any of the lactobacilli or bifidobacteria tested by Rodríguez et al. (2012), either isolated in that work or from commercial origin (Rodríguez et al. 2012).

No virulence factors were detected in INIA P708 by VirulenceFinder v2.0.4, nor by BLAST alignments of gene and protein sequences against the VFDB database. Pathogenicity values obtained by the machine learning model WSPC v0.0.6 and the PathogenFinder v1.1 web server were very low, and neither of the methods identified INIA P708 as a pathogenic strain. These results confirmed the QPS status of INIA P708. Similarly, no virulence factors or very low values of pathogenicity were previously shown for *L. paracasei* INIA P272 (Rodrigo-Torres et al. 2022).

Several putative bacteriophage sequences and defense systems were found in the INIA P708 genome. *Lactobacillus* phages iA2, PLE2, and PLE3 have also been identified in other commercial probiotic strains of the *Lacticaseibacillus* genus (Dieterle et al. 2016).

This CRISPR/Cas subtype II-A has also been reported in other strains of the same species (Pujato et al. 2021).

## Conclusions

The probiotic traits, technological properties, and the results derived from its genome analysis confirm *L. paracasei* INIA P708 identification and safety, support its use as an industrial strain, and reveal new potential probiotic traits in this strain. Further work is needed to confirm the *in vivo* probiotic properties of the INIA P708 strain. Future elucidation of the EPS structure by nuclear magnetic resonance would be of great help. In contrast to EPS species shed into the medium, INIA P708 EPS seems to be anchored to the cell wall. Purification to enable structural studies might thus require harsh extraction conditions such as acid extraction, which produce partial EPS fragmentation.

## Supplementary information

Below is the link to the electronic supplementary material.

ESM 1 (DOCX 41.0 MB)

## Data Availability

Sequence data were deposited at the NCBI under BioProject number PRJNA1401546. All data needed to evaluate the conclusions in this manuscript are presented in it and/or in the Supplementary materials.
