# Supplementary material for: Lacticaseibacillus paracasei INIA P708: a safe human isolate with probiotic and technological potential
Source: Appl Microbiol Biotechnol. 2026 Aug 4;110:330. doi: 10.1007/s00253-026-13984-x (PMC13724635; doi:10.1007/s00253-026-13984-x)
Supplement: Supplementary file 1 [file 253_2026_13984_MOESM1_ESM.docx]

Title: Functional analysis and closed genome sequence of *Lacticaseibacillus paracasei* INIA P708.

Journal: Applied Microbiology and Biotechnology

Authors: Eduardo D. Lozano-Escobar^1,2*+^, Eva Rodríguez-Mínguez^1*^, Karel H. M. van Wely^3^, Ángela Peirotén^1^, Modesto Redrejo-Rodríguez^2^, Antonia Picon^1^

Affiliations: ^1^ Departamento de Tecnología de Alimentos, Instituto Nacional de Investigación y Tecnología Agraria y Alimentaria (INIA), CSIC, Carretera de La Coruña Km 7, 28040, Madrid, Spain

^2^ Instituto de Investigaciones Biomédicas Sols-Morreale (UAM-CSIC), and Departamento de Bioquímica, Universidad Autónoma de Madrid (UAM), Arzobispo Morcillo 4 / Arturo Duperier 4, 28029, Madrid, Spain

^3^ Department of Immunology and Oncology, Centro Nacional de Biotecnología (CNB), CSIC Darwin 3, Campus UAM Cantoblanco, 28049, Madrid, Spain

* Both authors equally contributed to this work

^+^ Present adress: Instituto de Investigaciones Biomédicas Sols-Morreale (UAM-CSIC), and Departamento de Bioquímica, Universidad Autónoma de Madrid (UAM), Arzobispo Morcillo 4 / Arturo Duperier 4, 28029, Madrid, Spain

Corresponding authors: A. Picon, E-mail address: apicon@inia.csic.es; M. Redrejo-Rodríguez, E-mail address: modesto.redrejo@uam.es

Figure S1. Detection of the ropy phenotype in *L. paracasei* INIA P708 and *L. rhamnosus* GG.

Figure S2. Antimicrobial activity against pathogenic strains (*Clostridium perfringens* CECT 486; *Listeria monocytogenes* Scott A CECT 5672; *Klebsiella oxytoca* INIA Col 108; *Salmonella enterica* serovar Enteritidis CECT 4155) in BHI agar plates.

INIA P708 or GG cells grown on BHI plates were suspended in sterile 0.2% peptone water with 0.05% cysteine-HCl (approx. 8.5 log CFU / mL), and drops (5 µL) were deposited, in duplicate, on BHI agar plates. After anaerobic incubation at 37 °C for 24 h, a second layer of a 50 °C-tempered BHI soft agar, previously inoculated with fresh cultures of each pathogenic strain (at a 0.1%, final concentration of approx. 5.5-6 log CFU / mL), was poured. After incubation (24 h at 37 °C), plates were examined for the presence of inhibition halos.

Table S1. KEGG functional category distribution of functional annotation results and number of terms in the *L. paracasei* INIA P708 genome. The table below shows the 16 main categories.

| Functional category | no. |
| --- | --- |
| Carbohydrate metabolism | 252 |
| Energy metabolism | 66 |
| Lipid metabolism | 47 |
| Nucleotide metabolism | 66 |
| Amino acid metabolism | 101 |
| Metabolism of other amino acids | 35 |
| Glycan biosynthesis and metabolism | 46 |
| Metabolism of cofactors and vitamins | 71 |
| Metabolism of terpenoids and polyketides | 20 |
| Biosynthesis of other secondary metabolites | 37 |
| Xenobiotics biodegradation and metabolism | 21 |
| Genetic information processing | 167 |
| Environmental information processing | 223 |
| Cellular processes | 61 |
| Organismal systems | 38 |
| Human diseases | 64 |

Table S2. Distribution of CAZyme families annotated in the *L. paracasei* INIA P708 genome.

| CAZyme class | No. | Family code (no. genes) |
| --- | --- | --- |
| Glycoside hydrolases (GH) | 16 | GH3 (1), GH4 (1), GH13 (3), GH20 (1), GH29 (3), GH31 (1), GH32 (1), GH38 (1), GH65 (1), GH88 (1), GH13-GH31 (2) |
| Glycosyl transferase (GT) | 22 | GT1 (7), GT2 (2), GT4 (5), GT5 (1), GT28 (1), GT35 (1), GT51 (4), GT2-GT4 (1) |
| Carbohydrate-binding modules (CBM)-GH | 2 | CBM48-GH13, CBM48-GH13-GH31 |
| Carbohydrate esterases (CE) | 1 | CE1 (1) |
| Polysaccharide lyase (PL) | 1 | PL8 (1) |

Table S3. List of stressors / probiotic marker genes annotated in the *L. paracasei* INIA P708 genome.

| Category | Location | Gene | Description | copy no. |
| --- | --- | --- | --- | --- |
| Heat stress | INIAP708_13345 | *hslO* | Hsp33 family molecular chaperone HslO | 1 |
|  | INIAP708_08810 | *dnaJ* | Molecular chaperone DnaJ | 1 |
|  | INIAP708_08815 | *dnaK* | Molecular chaperone DnaK | 1 |
|  | INIAP708_08820 | *grpE* | Nucleotide exchange factor GrpE | 1 |
|  | INIAP708_08825 | *hrcA* | Heat-inducible transcriptional repressor HrcA | 1 |
|  | INIAP708_13265 | *ctsR* | Transcriptional regulator CtsR | 1 |
|  | INIAP708_08065 | *hslV* | ATP-dependent protease subunit HslV | 1 |
|  | INIAP708_08070 | *hslU* | ATP-dependent protease ATPase subunit HslU | 1 |
|  | INIAP708_11955 | *groES* | Co-chaperone GroES | 1 |
|  | INIAP708_11950 | *groL* | Chaperonin GroEL | 1 |
|  | INIAP708_07735 | *clpX* | ATP-dependent Clp protease ATP-binding subunit ClpX | 1 |
|  | INIAP708_07810 | *clpB* | ATP-dependent chaperone ClpB | 1 |
|  | INIAP708_05350 | *clpP* | ATP-dependent Clp endopeptidase proteolytic subunit ClpP | 1 |
|  | INIAP708_04320 | *ccpA* | Catabolite control protein A |  |
| Cold stress | INIAP708_0346, INIAP708_07480 | *cspC* | Cold shock protein, CspA family | 2 |
| Acid stress | INIAP708_08940 | *plsC* | 1-acyl-sn-glycerol-3-phosphate acyltransferase | 1 |
|  | INIAP708_07850 | *pyk* | Pyruvate kinase | 1 |
|  | INIAP708_06895 | *atpA* | F0F1 ATP synthase subunit alpha | 1 |
|  | INIAP708_06875 | *atpB* | F0F1 ATP synthase subunit A | 1 |
|  | INIAP708_06910 | *atpC* | F0F1 ATP synthase subunit epsilon | 1 |
|  | INIAP708_06905 | *atpD* | F0F1 ATP synthase subunit beta | 1 |
|  | INIAP708_06880 | *atpE* | F0F1 ATP synthase subunit C | 1 |
|  | INIAP708_06885 | *atpF* | F0F1 ATP synthase subunit B | 1 |
|  | INIAP708_06900 | *atpG* | F0F1 ATP synthase subunit gamma | 1 |
|  | INIAP708_06890 | *atpH* | ATP synthase F1 subunit delta | 1 |
| Bile | INIAP708_08110 | *pPX1* | Manganese-dependent inorganic pyrophosphatase | 1 |
| resistance | INIAP708_11315 | *cfa* | Cyclopropane fatty-acyl-phospholipid synthase and related methyltransferases | 1 |
|  | INIAP708_09265 | *glnA* | Type I glutamate--ammonia ligase | 1 |
|  | INIAP708_00845, INIAP708_07100 | *oppA* | ABC-type oligopeptide transport system, periplasmic component | 2 |
|  | INIAP708_10445 | *oppC* | Oligopeptide transport system permease protein oppC | 1 |
|  | INIAP708_10435 | *oppD* | Oligopeptide transport ATP-binding protein OppD | 1 |
|  | INIAP708_13820 | *lpd* | Dihydrolipoamide dehydrogenase (E3) component of pyruvate/2-oxoglutarate dehydrogenase complex or glutathione oxidoreductase | 1 |
|  | INIAP708_07925 | *rpsA* | 30S ribosomal protein S1 | 1 |
|  | INIAP708_08610 | *rpsU* | 30S ribosomal protein S21 | 1 |
|  | INIAP708_08925 | *rpsB* | 30S ribosomal protein S2 | 1 |
|  | INIAP708_09005 | *rpsP* | 30S ribosomal protein S16 | 1 |
|  | INIAP708_07815 | *rpmF* | 50S ribosomal protein L32 | 1 |
|  | INIAP708_08980 | *rplS* | 50S ribosomal protein L19 | 1 |
|  | INIAP708_09090 | *rpmB* | 50S ribosomal protein L28 | 1 |
|  | INIAP708_09230 | *rpmA* | 50S ribosomal protein L27 | 1 |
|  | INIAP708_09240 | *rplU* | 50S ribosomal protein L21 | 1 |
|  | INIAP708_09495 | *rplT* | 50S ribosomal protein L20 | 1 |
|  | INIAP708_09500 | *rpmI* | 50S ribosomal protein L35 | 1 |

|  | INIAP708_00550 | *rplI* | 50S ribosomal protein L9 | 1 |
| --- | --- | --- | --- | --- |
|  | INIAP708_02400 | *cbh* | Choloylglycine hydrolase | 1 |
|  | INIAP708_07605 | *pdhA* | Pyruvate dehydrogenase E1 component subunit alpha | 1 |
|  | INIAP708_15370 | *nagB* | Glucosamine-6-phosphate deaminase | 1 |
| Osmosis | INIAP708_00325 | *osmF* | Periplasmic glycine betaine/choline-binding (lipo)protein of an ABC-type transport system (osmoprotectant binding protein) | 1 |
|  | INIAP708_00335 | *opuBA* | ABC-type proline/glycine betaine transport system, ATPase component | 1 |
|  | INIAP708_00320, INIAP708_00330 | *opuBB* | ABC-type proline/glycine betaine transport system, permease component | 2 |
|  | INIAP708_11485 | *opuAC* | Glycine betaine ABC transport system, glycine betaine-binding protein OpuAC | 1 |
|  | INIAP708_11495 | *proV* | ABC-type proline/glycine betaine transport system, ATPase component | 1 |
|  | INIAP708_11490 | *proW* | ABC-type proline/glycine betaine transport system, permease component | 1 |
|  | INIAP708_04280 | *glpF* | Glycerol uptake facilitator protein | 1 |
| Adhesion | INIAP708_08330 | *lspA* | Signal peptidase II | 1 |
|  | INIAP708_07720 | *tuf* | Elongation factor Tu | 1 |
|  | INIAP708_02590 | *srtA* | Sortase (surface protein transpeptidase) | 1 |
|  | INIAP708_07650 | *pyc* | Pyruvate carboxylase | 1 |
|  | INIAP708_05625 | *tpiA* | Triose-phosphate isomerase | 1 |
|  | INIAP708_08020 | *gap* | Type I glyceraldehyde-3-phosphate dehydrogenase | 1 |
|  | INIAP708_05630 | *eno* | Phosphopyruvate hydratase | 1 |
|  | INIAP708_06405 | *pgi* | Glucose-6-phosphate isomerase | 1 |
| Gut | INIAP708_14955 | *celC* | PTS cellobiose-specific component IIA | 1 |
| persistence | INIAP708_01500, INIAP708_11165, INIAP708_14030, INIAP708_14940 | *celB* | PTS cellobiose-specific component IIC | 4 |
|  | INIAP708_09900 | *celB* | PTS cellobiose transporter subunit IIC | 1 |
|  | INIAP708_01505 | *celA* | PTS cellobiose-specific component IIB | 1 |
| Oxidative | INIAP708_04005 | *tpx* | Thiol peroxidase | 1 |
| stress | INIAP708_01295 | *trxA* | Thioredoxin | 1 |
|  | INIAP708_04235 | *trxA* | Thioredoxin | 1 |
|  | INIAP708_09585 | *trxA* | Thiol-disulfide isomerase or thioredoxin | 1 |
|  | INIAP708_05275 | *trxB* | Thioredoxin-disulfide reductase | 1 |
|  | INIAP708_12250 | *ndh* | NADH dehydrogenase, FAD-containing subunit | 1 |
|  | INIAP708_08370 | *nrdH* | Glutaredoxin | 1 |
|  | INIAP708_02475 | *ilvB* | Pyruvate oxidase | 1 |
|  | INIAP708_09920, INIAP708_11480 | *spxB* | Pyruvate oxidase | 2 |
|  | INIAP708_00195, INIAP708_12785 | *mntH* | Nramp family divalent metal transporter | 2 |
|  | INIAP708_07075, INIAP708_07995 | *msrA* | Peptide methionine sulfoxide reductase MsrA | 2 |
|  | INIAP708_08630 | *msrB* | Peptide methionine sulfoxide reductase MsrB | 1 |
|  | INIAP708_13215 | *ahpC* | Alkyl hydroperoxide reductase C | 1 |
| Inmuno- | INIAP708_04005 | *tpx* | Thiol peroxidase | 1 |
| modulation | INIAP708_01295 | *trxA* | Thioredoxin | 1 |
|  | INIAP708_04235 | *trxA* | thioredoxin | 1 |
|  | INIAP708_09585 | *trxA* | Thiol-disulfide isomerase or thioredoxin | 1 |
|  | INIA-P708_12655 | *comA* | Transport/processing ATP-binding protein ComA (*plnG*) | 1 |
|  | INIA-P708_12650 | *abpD* | AbpD bacteriocin export accessory protein | 1 |
|  | INIA-P708_03165 |  | PTS sugar transporter subunit IIA | 1 |
|  | INIA-P708_14130 |  | N-acetylgalactosamine permease IID component | 1 |
|  | INIA-P708_14135 |  | PTS sugar transporter subunit IIC | 1 |
|  | INIA-P708_14140 |  | N-acetylgalactosamine-specific phosphotransferase enzyme IIB component 2 | 1 |
|  | INIA-P708_01435 | *mngR* | DNA-binding transcriptional regulator, GntR family | 1 |
|  | INIA-P708_12600 | *xre* | Transcriptional regulator, Xre family | 1 |
| Metal | INIAP708_13620 | *czcD* | Co/Zn/Cd efflux system component | 1 |
| resistance | INIAP708_03985 | *corA* | CorA family magnesium/cobalt transporter | 1 |
|  | INIAP708_05095, INIAP708_10595 | *corA* | Mg^2+^ and Co^2+^ transporter CorA | 2 |
|  | INIAP708_13620 | *czcD* | Co/Zn/Cd efflux system component | 1 |

Abbreviations: ABC = ATP binding cassette; PTS = Phosphotransferase system
